# Editorial: Underlying molecular interconnections of the estrogen receptor alpha and associated factors involved in breast cancer development: the way to new therapeutic approaches, volume II

**DOI:** 10.3389/fendo.2024.1427468

**Published:** 2024-06-14

**Authors:** Philipp Y. Maximov, Guy Leclercq

**Affiliations:** ^1^ JSC Generium, Moscow, Russia; ^2^ Institute Jules Bordet, Faculty of Medicine, Université Libre de Bruxelles, Brussels, Belgium

**Keywords:** estrogen receptor alpha, transcription, coregulators, breast cancer, endocrine dependency, endocrine treatment resistance, therapeutic perspectives

Estrogen Receptor alpha (ERα) belongs to a class of ligand- dependent nuclear receptors functioning as transcription factors that contribute to the development of the mammary gland and its unfortunate neoplastic transformation. This phenomenon is partly the result of a cooperation between estrogen production and a multitude of other extracellular signals (hormonal, immunological, calcium variation…) (1–3). Therefore, ERα can be viewed as a hub in which the incorrect processing of these signals contributes to tumorigenesis preceding its progressive evolution to resistance to antiestrogens (tamoxifen and related SERMs) in addition to inhibitors of estrogen biosynthesis (aromatase inhibitors). The administration of low doses of estrogens or receptor degraders (SERDs), which induce apoptosis in the tumor (4), represents an alternative approach that identifies ERα as a therapeutic target.

Our Research Topic is dedicated to the search for novel approaches to overcome this endocrine resistance. Its first volume describes some aspects that could be taken into account as therapeutic approaches; regulation of the expression of the receptor (turnover, successive transient conformational changes implicated in the transport to the nucleus, homo dimerization of the receptor required for its association with the palindromic sequence of nucleotides of the DNA localized in the promoters of the genes to be transcribed, as well as heterodimerization of the ERα with another transcription factor with which it associates as a co-regulator). This large number of potential therapeutic approaches suggests a myriad of underlying therapeutic possibilities. A view that is largely confirmed by the investigations collected in this second volume.

Kiliti et al. provides a detailed overview of the AIB1 co-activator as an example. AIB1 is overexpressed in a subset of breast cancers associated with a poor prognosis. The authors describe its hormone-independent mechanism of action, and its role as an oncogene interacting with other nuclear receptors, in particular AR (also a known factor in breast cancer development). This status would logically derive from the high plasticity of its ERα recruitment region where the anchorage LxxLL motif would not be stabilized in conformation to satisfy its transient function. A property that in fact concerns all coregulators. Seeking to stabilize the exposure of such a coregulator recruitment region in a drug design program represents obviously a time–consuming enterprise, a factor that may explain the relative lack of relevant publications on this subject.

This pessimistic view has led the scientific community to shift their focus to curative approaches without addressing direct/stable interactions between ERα and its coregulators. The knowledge that glucose starvation enhances the antiproliferative and antiestrogenic potency of hormone–dependent breast cancer cells provides hope in the blockade of metabolic energy (ATP) production for such a task (5) since the dynamic of coregulator exposure is highly energy-dependent (6, 7).

This approach, which is not restricted to glycolysis but also to a large panel of metabolic pathways regulating energy production (8, 9), led Cipolletti et al. to start an investigation on GART, an enzyme for the *de novo* purine metabolic pathway whose expression correlates with longer relapse-free survival in breast cancer patients. Using ERα-positive breast cancer cells, the authors identified lometrexol, a GART inhibitor, as an ER that exerts an antiproliferative effect, even in tamoxifen-resistant cells, similar to that of fulvestrant, a SERD; a synergistic effect with the CDK4/CDK6 inhibitors was also observed. Therefore, targeting GART for treatment at different stages of the disease may be plausible.

Complementary to these investigations, we received four clinical reports from Chinese Institutions revealing a local interest in our therapeutic approach. Two of them were accepted. Wang et al. delivered an exhaustive panoramic review of the worldwide literature on endocrine therapy including an evaluation of drug efficacy, molecular markers, and receptor interactions, providing guidelines for further investigations. Zhang et al. provided a case report of a neoadjuvant combination of concomitant endocrine therapy and chemotherapy in a patient with locally advanced breast cancer unresponsive to two prior chemotherapies. In this patient, the switch to combination therapy with fulvestrant and capecitabine resulted in tumor regression without adverse events, suggesting that this unexplored approach may be appropriate for ER-positive cancers in a locally advanced clinical setting. The information contained in these publications may also be of interest to breast medical oncologists.

## Author‘s note

GL initiated this publication project in collaboration with Filippo Acconcia as co-Editor. Seven accepted contributions were reported in the first Volume in 2021. The follow-up to this project was conducted with PM who managed all received manuscripts except for the contribution by F. Acconcia, which justified its first authorship. GL was responsible for manuscript writing under a conceptual agreement.

## Author contributions

PM: Writing – review & editing. LG: Writing – review & editing, Writing – original draft.
